# Successful Treatment of Adalimumab-Induced Paradoxical Skin Reactions in Pustulotic Arthro-Osteitis With Guselkumab

**DOI:** 10.7759/cureus.70321

**Published:** 2024-09-27

**Authors:** Kazuki Yatsuzuka, Takuya Matsumoto, Teruki Kidani, Yasuhiro Fujisawa, Masamoto Murakami

**Affiliations:** 1 Department of Dermatology, Ehime University Graduate School of Medicine, Toon, JPN; 2 Department of Hematology, Clinical Immunology, and Infectious Diseases, Ehime University Graduate School of Medicine, Toon, JPN; 3 Department of Bone and Joint Surgery, Ehime University Graduate School of Medicine, Toon, JPN; 4 Department of Anatomy, Miyazaki University, Miyazaki, JPN

**Keywords:** adalimumab, alopecia, palmoplantar pustulosis, psoriasis, pustulotic arthro-osteitis

## Abstract

Pustulotic arthro-osteitis (PAO) is a significant comorbidity of palmoplantar pustulosis (PPP), with biologics targeting tumor necrosis factor (TNF)-α, interleukin (IL)-12/23 p40, IL-23 p19, and IL-17 showing clinical benefits for PPP/PAO. However, patients receiving these biological agents frequently experience paradoxical skin reactions (PSRs), particularly with anti-TNF-α treatments. We report a case of PPP/PAO treated with the anti-TNF-α agent adalimumab, which led to the development of PSRs, including psoriasis-like and folliculitis-like rashes, and acute hair loss. Subsequently, treatment was changed to guselkumab, an anti-IL-23 p19 monoclonal antibody, which successfully controlled both PPP/PAO and PSRs. To date, no PSRs associated with anti-IL-23 agents in PAO have been reported. A study from Japan indicates that guselkumab and adalimumab have similar efficacy in treating PAO. Given that anti-IL-23 agents are approved for refractory PPP under the Japanese health insurance system, we recommend their use over adalimumab in PPP/PAO patients to prevent PSRs.

## Introduction

Palmoplantar pustulosis (PPP) is a chronic pustular dermatitis affecting the palms and soles, characterized by vesicles, pustules, erythema, and abnormal desquamation [1]. It is recognized as a common skin disease in Japan [1]. There is continuing controversy over whether PPP is a psoriasis-related disease. Our group proposed that PPP should be subdivided into types A, a rare association with plaque-type psoriasis, and B, frequently associated with plaque-type psoriasis [1]. In type A-PPP, vesicles appear before pustules, whereas type B-PPP lacks vesicles [1]. Most PPP in Japan are consistent with type A-PPP. Hence, in this article, we described about type-A PPP. Its pathomechanism remains unclear, but the analysis of the serum and skin lesions of PPP patients has demonstrated that the Th17/interleukin (IL)-23 pathway is important in PPP [2]. More importantly, the most common site of PPP development is around the acrosyringium. The eccrine sweat-derived antimicrobial peptides (hCAP-18/LL-37) were detected in the early vesicles of PPP [1], and we also showed a direct relationship between the acrosyringium and vesicles/pustules of PPP using two-photon excitation microscopy [3]. In addition, we recently demonstrated that partial damage of the acrosyringium in the palms and soles causes IL-1-rich eccrine sweat to leak into the epidermis and IL-1 disrupts E-cadherin expression on keratinocytes, leading to intraepidermal vesicle formation in a PPP mouse model [4]. Pustulotic arthro-osteitis (PAO), a significant comorbidity of PPP, affects joints, causing pain and deformity. It primarily impacts the clavicles, sternum, and sternoclavicular joints, though peripheral joints may also be involved [5]. Approximately 20-30% of PPP patients develop PAO, as reported in previous studies [5]. Although its pathogenesis is not well understood, considering the association between PPP and PAO, IL-23-mediated inflammation may be involved in causing PAO. In addition, a study by Ueno et al. demonstrated the proportion of activated Th17 cells was significantly higher in the peripheral blood of patients with PAO than in healthy controls [6]. Treatment options include topical corticosteroids, vitamin D3, phototherapy, retinoids for PPP, systemic corticosteroids, non-steroidal anti-inflammatory drugs (NSAIDs), methotrexate for PAO, cyclosporine, smoking cessation, and managing focal infections such as tonsillitis, sinusitis, and dental infections for PPP/PAO. Recent research highlights the effectiveness of biologics, such as anti-tumor necrosis factor (TNF)-α, IL-12/23 p40, IL-23 p19, and IL-17, in treating PPP/PAO [5]. Consequently, biological agents are increasingly used for refractory PPP/PAO in Japan. However, these agents may induce paradoxical skin reactions (PSRs), which are new or exacerbated immune-mediated skin disorders triggered by the targeted treatment, especially with anti-TNF-α agents [7,8]. Here, we describe a case of adalimumab-induced PSRs in a PPP/PAO patient, successfully managed with guselkumab, and discuss the sequencing of biologics in treating PPP/PAO.

## Case presentation

**Figure 1:**
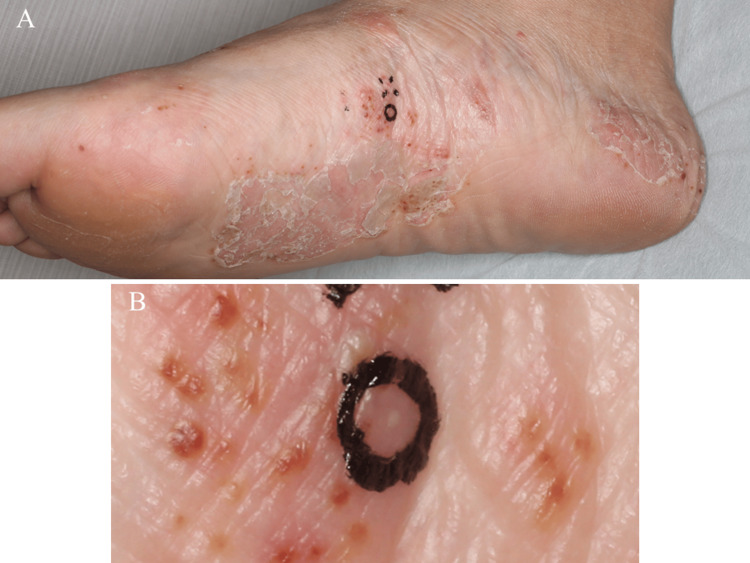
Cutaneous findings of palmoplantar pustulosis During the first visit to our department, multiple scaly erythemas, vesicles, pustulo-vesicles, and pustules were seen on the soles (A, B).

**Figure 2:**
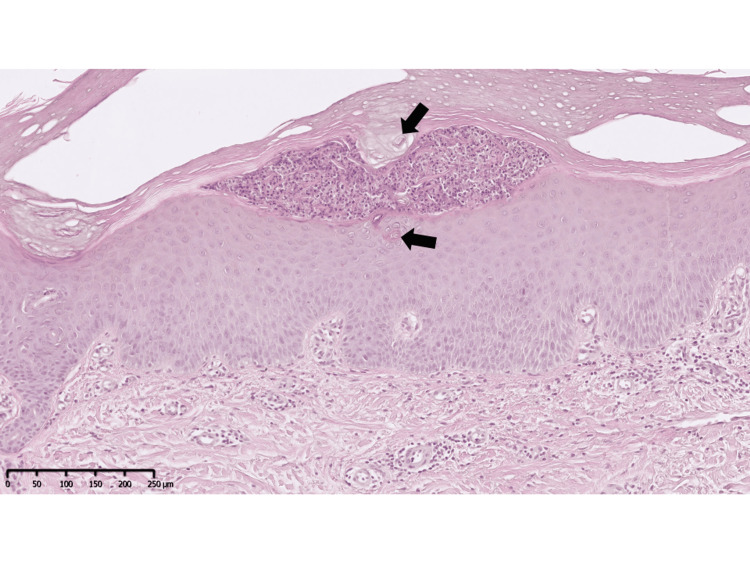
Histological findings (hematoxylin and eosin stain) of palmoplantar pustulosis A skin biopsy from a pustule on the sole revealed a subcorneal pustule filled with abundant neutrophils close to the acrosyringium (arrow). The scale bar is 250 μm.

A 51-year-old woman presented to our department with a five-month history of multiple scaly erythemas, vesicles, pustulo-vesicles, and pustules on her palms and soles (Figure 1A). Concurrently, she experienced severe pain in her sternoclavicular joint and left thigh. She had a history of Hashimoto's disease, for which she was taking oral levothyroxine sodium hydrate, and had suffered from tonsillitis in her 30s as a focal infection. She was also a current smoker. A skin biopsy from a pustule on her sole (Figure 1B) revealed a subcorneal pustule abundant in neutrophils close to the acrosyringium (Figure 2), consistent with PPP.

**Figure 3:**
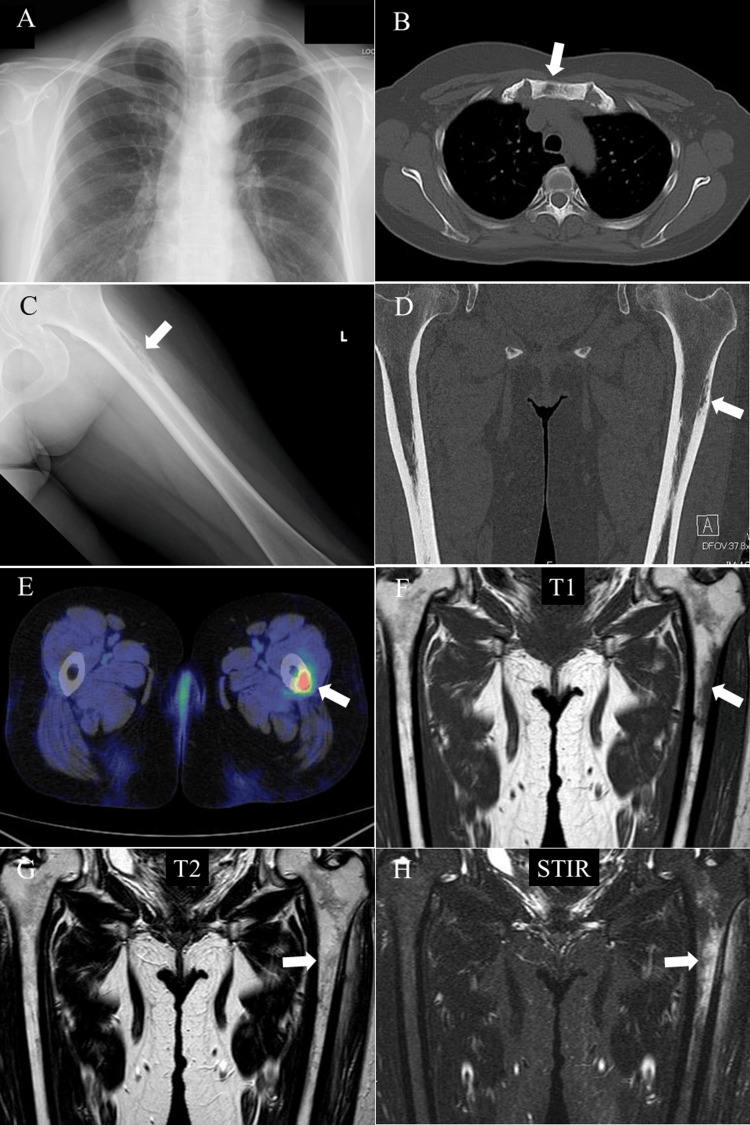
Radiological findings of pustulotic arthro-osteitis (A) Chest X-ray, (B) chest CT, (C) X-ray of the left thigh, (D) CT of the thighs, (E) positron emission tomography-CT of the thighs, (F) coronal T1-weighted MRI of the thighs, (G) coronal T2-weighted MRI of the thighs, and (H) coronal STIR-weighted MRI of the thighs. Each arrow indicates the bone lesions. CT: computed tomography; MRI: magnetic resonance image; STIR: short tau inversion recovery

Chest X-ray and computed tomography (CT) showed bone sclerosis in the sternum (Figure 3A, 3B). X-ray of the left thigh showed a clear bone image on the proximal lateral side of the femur (Figure 3C), and CT revealed thickening of the bone cortex in the same area with a mild periosteal reaction (Figure 3D). Positron emission tomography-CT indicated mild uptake around the sternocostoclavicular joints and strong uptake (SUVmax=6.8) in the bone cortex of the proximal part of the left femoral shaft (Figure 3E). Magnetic resonance imaging of the left thigh showed edema-like abnormal signals in the proximal femoral shaft bone cortex (Figure 3F-3H).

**Figure 4:**
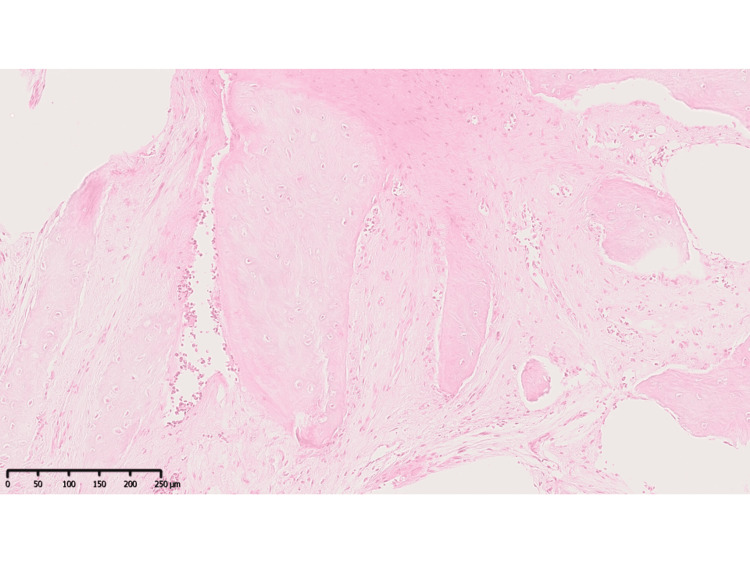
Histological findings (hematoxylin and eosin stain) of pustulotic arthro-osteitis A bone biopsy from the left thigh revealed osteitis fibrosa without neoplastic changes. The scale bar is 250 μm.

Given these findings, the lesion in the left femur was suspected to be chronic osteomyelitis or a bone tumor, including bone metastasis. A subsequent bone biopsy revealed osteitis fibrosa (Figure 4), leading to a diagnosis of PPP/PAO.

**Figure 5:**
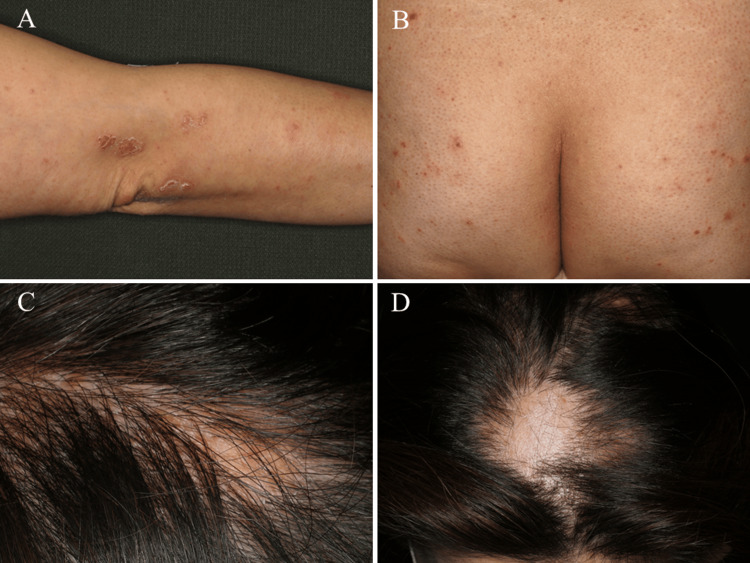
Cutaneous findings of paradoxical skin reactions (A) Psoriasis-like rash, (B) folliculitis-like rash, and (C, D) acute hair loss.

For treatment, we initially recommended smoking cessation and administered topical steroids, vitamin D3, and NSAIDs for joint pain. However, these measures were unsuccessful, and she gradually began experiencing difficulty walking. Unable to obtain consent for a tonsillectomy, we prescribed adalimumab and 4 mg/week of methotrexate for the joint pain; we selected a combination of these two drugs rather than a higher dose of methotrexate or anti-IL-23 agents due to the severe pain, resulting in symptom improvement within a month. However, five months later, she developed a psoriasis-like rash (Figure 5A) and folliculitis-like rash (Figure 5B) on her extremities and trunk, along with acute hair loss (Figure 5C, 5D).

**Figure 6:**
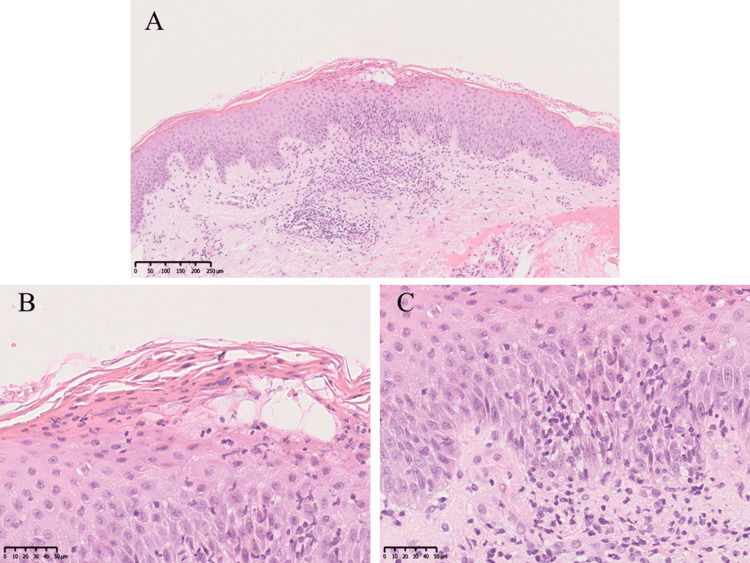
Histological findings (hematoxylin and eosin stain) of paradoxical skin reactions A skin biopsy from a psoriasis-like rash on the forearm. (A) At the low-power magnification, hyperkeratosis and acanthosis were seen. (B, C) At the high-power magnification, parakeratosis, disappearance of the granular layer, neutrophil and lymphocyte infiltration into the epidermis with focal vacuolization, and infiltration of lymphocytes into the dermis were seen. The scale bars are 250 μm in (A) and 50 μm in (B) and (C).

A skin biopsy from a scaly erythema on the forearm revealed hyperkeratosis, parakeratosis, acanthosis, disappearance of the granular layer, infiltration of neutrophils and lymphocytes into the epidermis with focal vacuolization, and infiltration of lymphocytes into the dermis (Figure 6A-6C).

These findings led to a diagnosis of adalimumab-induced PSRs in PAO. In this case, despite the skin lesions of PPP tending to improve with adalimumab, new skin lesions occurred in conjunction with acute hair loss; hence, extra-palmoplantar lesions were ruled out. In addition, interface dermatitis and neutrophil infiltration under the stratum corneum are generally not observed in extra-palmoplantar lesions. We subsequently switched from adalimumab to guselkumab and discontinued methotrexate. The rash and hair loss gradually improved and entirely resolved after three months and 10 months after the switch, respectively. Currently, three years have passed since initiating guselkumab treatment, there is no recurrence of PSRs, the joint pain remains in remission, and the skin lesions of PPP leave a slight keratinization on the soles.

## Discussion

PSRs are frequently reported in patients treated with biologics for conditions like rheumatoid arthritis, Crohn's disease, ulcerative colitis, and ankylosing spondylitis. PSRs manifest in various forms including psoriasiform dermatitis, eczema, lupus-like reactions, sarcoidosis-like reactions, alopecia, and PPP-like pustulosis [7,8]. Though rare, folliculitis-like eruptions similar to our case have been documented [8]. PSRs occur at varying frequencies depending on the underlying condition, with reports of 29% in inflammatory bowel disease (IBD) [7]. Among biologics, anti-TNF-α agents are most commonly associated with PSRs [8,9]. PSRs have also been observed in dermatologic conditions treated with biologics, such as psoriasis, psoriatic arthritis, and PPP [8]. The mechanism behind PSRs is unclear and may vary by drug. One theory suggests that anti-TNF-α agents cause psoriasiform dermatitis due to attenuated TNF-α-dependent negative feedback on plasmacytoid dendritic cells, resulting in an overproduction of type I interferons [8]. Anti-IL-12/23 p40 agents may induce psoriasiform dermatitis by blocking both IL-23 and IL-12 pathways, which leads to the overexpression of IFN-α [10]. It is hypothesized that the blockade of IL-17A by anti-IL-17 agents may cause compensatory overproduction of cytokines (IL-23, IL-17F, IL-12, and TNF-α) [10]. Management strategies for PSRs vary with severity; mild to moderate cases may continue biologics alongside topical, physical (phototherapy), or systemic therapies (methotrexate, mycophenolate mofetil, or acitretin). Moderate to severe cases generally require discontinuation of the offending biologic and initiation of a different drug class [8]. For severe PSRs, 11% of IBD patients reportedly discontinued biologics [7].

In our case, the patient with PPP/PAO initially responded to adalimumab but developed PSRs, including psoriasiform dermatitis, folliculitis-like eruptions, and acute hair loss, prompting discontinuation due to severe hair loss. Numerous PAO cases treated with anti-TNF-α agents have reported similar PSRs [11]. However, to our knowledge, no PSRs with anti-IL-23 agents have been reported in PAO. Guselkumab, an anti-IL-23 agent, has proven effective for joint symptoms among Japanese PPP/PAO patients [12]. Furthermore, a study by Ueno et al. demonstrated comparable efficacy between guselkumab and adalimumab in Japanese PAO patients [6]. With anti-IL-23 agents (guselkumab and risankizumab) approved for refractory PPP in Japan, we opted to switch from adalimumab to guselkumab, resulting in an improvement in PSRs and sustained control of PPP/PAO. Thus, we propose that anti-IL-23 agents may be a better option than anti-TNF-α agents for patients with PPP/PAO.

## Conclusions

This case highlights a potentially significant association between anti-TNF-α agents and PSRs in a patient with PPP/PAO. Our patient developed a psoriasis-like rash, folliculitis-like rash, and acute hair loss after initiating adalimumab. To our knowledge, no PSRs with anti-IL-23 agents have been reported in PAO patients. Hence, we changed to guselkumab, which successfully controlled both PPP/PAO and PSRs. Given the similar efficacy of anti-IL-23 agents in PAO patients and their potentially lower risk of PSRs, we recommend using anti-IL-23 agents over anti-TNF-α agents for managing PAO.
